# A novel *Bacillus megaterium*/ω-3 PUFA synbiotic attenuates colon inflammation and promotes a pro-resolving macrophage profile ex-vivo

**DOI:** 10.3389/fimmu.2026.1916994

**Published:** 2026-08-25

**Authors:** Valeria Prete, Francesca Picone, Angela Carmelita Abate, Bodo Speckmann, Thomas Berngruber, Concetta Iside, Eleonora Venturini, Carmine Vecchione, Carolina Ciacci, Albino Carrizzo

**Affiliations:** 1 Department of Medicine, Surgery and Dentistry, “Scuola Medica Salernitana” University of Salerno, Baronissi, Italy; 2 Department of Biology, University of Naples Federico II, Complesso Universitario Monte S. Angelo, Naples, Italy; 3 Evonik Operations GmbH, Hanau, Germany; 4 Kerry Global Innovation Centre, Naas, Ireland; 5 Vascular Physiopathology Unit, IRCCS Neuromed, Pozzilli, Italy

**Keywords:** *Bacillus megaterium*, intestinal bowel disease (IBD), macrophage polarization, specialized pro-resolving mediators, synbiotics, ulcerative colitis

## Abstract

**Introduction:**

Nutritional supplementation with ω-3 polyunsaturated fatty acids has shown potential benefits in inflammatory bowel disease, although clinical responses remain inconsistent. This may be partly due to the limited conversion of ω-3 PUFAs into specialized pro-resolving mediators under physiological conditions.

**Methods:**

We investigated whether fermentation of ω-3 PUFA lysine salt with Bacillus megaterium DSM 32963 enhanced its anti-inflammatory and pro-resolving activity. The resulting formulation was evaluated in ex vivo colon biopsies obtained from patients with ulcerative colitis (UC) and healthy subjects. Inflammatory cytokine expression and macrophage-associated markers were assessed using qPCR, Western blotting and immunofluorescence.

**Results:**

The B. megaterium/ω-3 PUFA formulation modulated the inflammatory response in ulcerative colitis biopsies, reducing the expression of selected pro-inflammatory cytokines and promoting a macrophage profile associated with resolution of inflammation.

**Discussion:**

These findings provide preliminary evidence that microbial conversion of ω-3 PUFAs can enhance their pro-resolving activity in human colonic tissue. This synbiotic approach may represent a promising nutritional adjunct for ulcerative colitis, although further in vivo and clinical investigations are required.

## 1 Introduction

Uncontrolled chronic inflammation is a pathogenic hallmark of many systemic conditions, including well-known inflammatory disorders such as arthritis, neurodegenerative diseases, asthma, cancer, metabolic syndrome, diabetes, autoimmune diseases, cardiovascular diseases and inflammatory bowel diseases (IBD) (1). The latter is a chronic condition encompassing ulcerative colitis (UC), Crohn’s disease (CD), and indeterminate colitis, all of which involve chronic inflammation of the intestinal tract, often with evidence of increased systemic inflammation (2).

IBD currently affects 6.8 million people worldwide, with the highest prevalence generally observed in Western countries, while rising incidence has also been reported in several regions of China, Asia, South America, and Africa (3, 4).

Large meta-analyses of cohort studies have reported associations between IBD and coronary heart disease (CHD) or atherosclerotic cardiovascular disease events (5). In recent years, the pathogenesis of IBD has been attributed to disturbances in both the immune and non-immune systems, or to imbalanced interactions with microbes. Growing evidence suggests that defects in pro-resolving pathways might strongly contribute to IBD onset and progression (6).

In this context, macrophages, the predominant mononuclear phagocyte in intestinal lamina propria, play a pivotal role in IBD control. Following microenvironmental changes, macrophages are polarized into two phenotypes named M1 pro-inflammatory or M2 anti-inflammatory macrophages (7). Recent evidence demonstrates that in experimental models of induced colitis and in IBD patients, the M1 macrophage population increases, while the M2 population decreases (8). This shift supports the hypothesis that a polarization imbalance (M1/M2 ratio) is a primary driver of disease progression. Salicylates and corticosteroids have been the first line of treatment for IBD due to their ability to manage pain and inflammation (9). Unfortunately, they do not change the natural history of the disease and are associated with significant adverse effects during long-term therapy (10).

The scientific community has drawn attention to the anti-inflammatory effects of ω-3 polyunsaturated fatty acids (PUFAs) found in oils derived from fish, seeds and microalgae, recommending them as dietary supplements to counteract various chronic inflammatory processes (11–13) and for their beneficial local action in the intestinal tract (14). Unfortunately, subsequent meta-analyses and double-blind RCTs have demonstrated that blood docosahexaenoic acid (DHA) and eicosapentaenoic acid (EPA) levels poorly correlate with reducing inflammatory risk and remain inconsistent and inconclusive to recommendations (15). The mixed efficacy of EPA and DHA supplementation may be attributable to differences in baseline and background intake of ω-3 PUFAs, their dosage, form and type of formulation, as well as intrinsic factors like gender, health status, and age.

The discovery of a new class of lipid mediators, called specialized pro-resolving mediators (SPMs), has highlighted the therapeutic benefits of PUFAs. SPMs are bioactive lipid mediators derived from ω-3 PUFAs that promote the resolution of inflammation by activating self-limiting host responses, including modulation of macrophage activation toward M2-like or pro-resolving-associated phenotypes (16). Various SPM families have been identified, endogenously derived from ω-6 arachidonic acid (AA), as well as ω-3 docosapentaenoic acid (DPA), DHA, and EPA (17). The SPMs’ families include E- and D- series resolvins, protectins, maresins, and lipoxins, which are produced and stimulated by endogenous enzymes using PUFAs as substrates (17). Each SPM has a unique structure, receptor, and mechanism of action, with most of them having a pro-resolving and anti-inflammatory effect (18, 19). Notably, unlike PUFAs, for which the therapeutic dose is in the range of 250 to ~4,000 mg, SPMs exert their effects at picomolar or nanomolar levels (16, 20) thus making them very attractive for therapeutic applications.

Furthermore, recent evidence indicates that the species *Bacillus megaterium* can catalyze the oxygenation of ω-3 PUFA into SPMs (21–23). This confirms that bacterial enzymatic conversion of fatty acids is a promising strategy for *in situ* production of bioactive lipid metabolites. In this regard, a pilot study and a subsequent controlled study conducted in healthy subjects with SynΩ3 have clearly demonstrated the capability to increase SPMs levels *in vivo*, thereby supporting its characterization as a self-sufficient SPM-producing formulation (23).

These findings prompted us to investigate the biological activity of an *in vitro*-generated, SPM- formulation in ex vivo colonic biopsies from patients with UC. Specifically, we evaluated its ability to modulate pro-inflammatory cytokine expression and macrophage-associated marker profiles. Our results provide preliminary evidence that this bioactive strategy can attenuate mucosal inflammatory responses and support its further investigation as a potential nutritional adjunct for UC.

## 2 Materials and methods

### 2.1 Fermentation of ω-3 fatty acids by *Bacillus megaterium* DSM 32963

To prepare dispersions of ω-3 fatty acids 0.8 g of dioleylphosphatidylcholine (DOPC, Lipoid GmbH) were dissolved in 1 ml of ethanol and 0.2 g of ω-3 lysine salt (AvailOm^®^, Evonik Essen, Germany) was added and dissolved. 1 ml of the respective solutions was added dropwise to 20 ml of a 0.1 M phosphate buffer, pH = 8, at a temperature of 45 °C under intense stirring. Afterward, the dispersion was put on ice and sonified for 15 minutes to generate nanometer-scale dispersions (Branson Sonifier, 100% amplitude, 50% impuls). Finally, the dispersion was sterile filtered through 0.2 pm syringe filters.

To produce Bacillus ferment, the dispersion was added to exponentially growing B. megaterium DSM 32963 cell cultures to a final concentration of 0.4 g/l of omega-3 lysine salt (AvailOm^®^ by Evonik) and incubated in Luria Bertani broth (LB, Thermo Fisher Scientific) with 0.1% Glucose (LBG) in 100 ml shaking flasks for 16 h at 30 °C and 200 rpm. Finally, the ferment was sterile filtered through 0.2 pm syringe filters.

As a sterile control the omega-3 lysine salt dispersion was added to sterile LBG medium, incubated sterile for 16 h at 30 °C and 200 rpm, and sterile filtered through 0.2 pm syringe filters.

Sterile filtered crude liquid from the Bacillus Ferment and Sterile Control were added to 5 ml buffer for biopsies at 1, 2.5, 5 or 10µL equivalent to dilutions of 5000, 2000, 1000 or 500-fold.

### 2.2 Lipid mediator metabololipidomics by UPLC-MS-MS

Lipid mediator (LM) analysis using ultra performance liquid chromatography-tandem mass spectrometer (UPLC-MS/MS) was performed as described previously with some minor modifications (24). Briefly, freshly thawed human plasma or *Bacillus megaterium* cultures were first mixed with the same volume of ice-cold methanol containing deuterium labeled internal standards (200 nM d8–5S-HETE, d4-LTB4, d5-LXA4, d5-RvD2, d4-PGE2 and 10 µM d8-AA; Cayman Chemical/Biomol GmbH, Hamburg, Germany) to facilitate quantification and sample recovery. Samples were kept at −20 °C for 60 min to allow protein precipitation. After centrifugation (1200× g, 4 °C, 10 min), 8 mL of acidified water was added (final pH = 3.5), and the samples were subjected to solid phase extraction (SPE). The SPE cartridges (Sep-Pak^®^ Vac 6cc 500 mg/6 mL C18; Waters, Milford, MA, USA) were equilibrated with 6 mL methanol and then with 2 mL water prior to sample loading onto the columns. After washing with 6 mL water and an additional 6 mL n hexane, LM were eluted with 6 mL methyl formate. The eluates were brought to dryness using a TurboVap LV evaporation system (Biotage, Uppsala, Sweden) and resuspended in 100 µL methanol/water (50/50, v/v) for analysis by UPLC-MS-MS. The LM were analyzed with an Acquity™ UPLC system (Waters, Milford, MA, USA) and a QTRAP 5500 Mass Spectrometer (ABSciex, Darmstadt, Germany), equipped with a Turbo V™ Source and electrospray ionization. LM were separated using an ACQUITY UPLC^®^ BEH C18 column (1.7 µm, 2.1 × 100 mm; Waters, Eschborn, Germany) at 50 °C with a flow rate of 0.3 mL/min and a mobile phase consisting of methanol/water/acetic acid of 42/58/0.01 (v/v/v) that was ramped to 86/14/0.01 (v/v/v) over 12.5 min and then to 98/2/0.01 (v/v/v) for 3 min (25). The QTrap 5500 was operated in negative ionization mode using scheduled multiple reaction monitoring (MRM) coupled with information-dependent acquisition. The scheduled MRM window was 60 sec, optimized LM parameters were adopted, and the curtain gas pressure was set to 35 psi. The retention time and at least six diagnostic ions for each LM were confirmed by means of external standards (Cayman Chemical/Biomol GmbH, Hamburg, Germany). Quantification was achieved by calibration curves for each LM. Linear calibration curves were obtained for each LM and gave r2 values of 0.998 or higher (for PUFA 0.95 or higher). Additionally, the limit of detection for each targeted LM was determined (25).

### 2.3 Participants

Treatment-naïve ulcerative colitis (UC) biopsy samples were obtained from patients managed at the U.O.C. of Gastroenterology of the A.O.U. San Giovanni di Dio e Ruggi d’Aragona (Salerno, Italy). The study protocol was approved by the Inter-Institutional Ethics Committee ‘Campania Sud’ (Comitato Etico Inter-Aziendale Campania Sud) under protocol number 0238828 (03/12/2021). Eligible participants were adults aged ≥18 years, including asymptomatic individuals or those with suspected ulcerative colitis. Healthy controls were asymptomatic adult volunteers with no history of chronic IBD or related conditions. Participants enrolled in the study were identified during a colonoscopy exam and provided with a participant information sheet. All participants provided written informed consent before inclusion. Information about demographics (sex, age and smoking status) was collected, and the Montreal classification was used to classify patients according to their UC phenotype. Furthermore, neither the patients with newly diagnosed ulcerative colitis nor the healthy subjects in the control group were taking any kind of medication, such as corticosteroids or azathioprine. All biopsies were taken from the recto-sigmoid portion of the colon. The baseline characteristics of patients with UC and healthy subjects are shown in **Table 1**.

**Table 1:** Baseline demographics and clinical phenotype of treatment- naïve ulcerative colitis patients (UC) and healthy controls.

Characteristics	Treatment-naïve ulcerative colitis (n = 13)	Healthy controls (n = 6)
Male sex, n (%)	7 (53,8%)	4 (67%)
Age median (range)	64,1 (29–91)	66,3 (38–84)
Smokers, n (%)		
Never	3 (23%)	3 (50%)
Current	6 (46,1%)	1 (16,7%)
Former	4 (30,7%)	2 (33,3%)
Extent, UC		
E1	13	
Treatment		
Corticosteroids	0	0
Azathioprine	0	0
Biopsy location	Recto-sigmoid	Recto-sigmoid

### 2.4 Human colon biopsies collection, culture and storage

Each participant’s colon biopsies were sampled, and the time and site data were recorded for each sample. After obtaining written informed consent from each donor, colon biopsies were collected from healthy subjects and patients with UC during colonoscopy. Multiple biopsies were randomly collected from endoscopically normal rectosigmoid mucosa in healthy controls and from endoscopically non-inflamed rectosigmoid mucosa adjacent to inflamed areas in treatment-naïve patients with UC.

**Figure 1:**
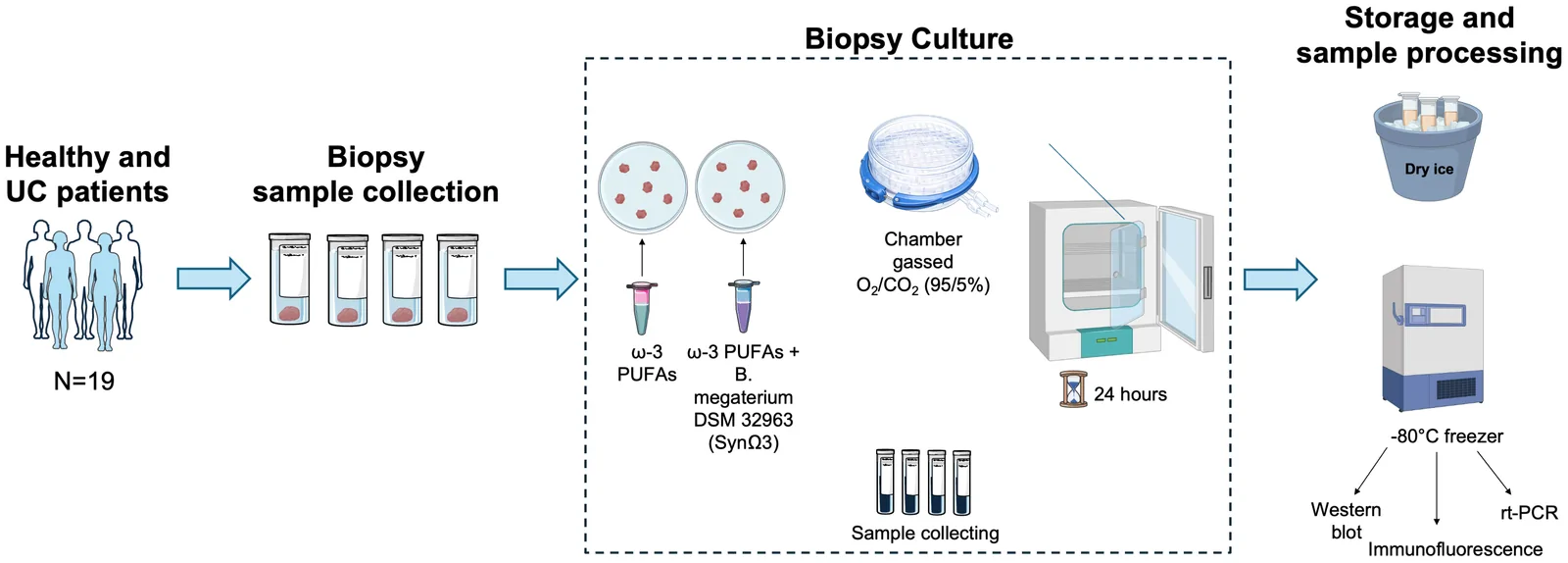
Study design and workflow for biological sample collection and processing.

Endoscopy procedures were performed at the University Hospital “San Giovanni di Dio e Ruggi d’Aragona” of Salerno as part of planned hospital care with consent to obtain additional biopsies for research. Each participant’s colon biopsies were sampled and the time and site data were recorded for each sample. After obtaining written informed consent from each donor, colon biopsies were collected from healthy subjects and patients with UC during colonoscopy. Three biopsies were randomly collected from endoscopically normal rectosigmoid mucosa in healthy controls and from endoscopically non-inflamed rectosigmoid mucosa adjacent to inflamed areas in patients with UC. For each participant (6 healthy controls and 13 patients with UC), the three biopsy specimens were each divided into two fragments, yielding six tissue fragments per participant, which were then randomly allocated to the experimental conditions. Fragments were immediately put in 0.9% NaCl and placed on a stainless-steel mesh over the central well of an organ culture dish containing RPMI culture medium supplemented with L-glutamine 2 Mm, 10% inactivated FBS, 1% PenStrep, with the epithelium facing upward. Colon biopsies were cultured with only medium (blank) and with SPMs at different concentrations (10 μl, 5 μl, 2,5 μl, 1,25 μl). The dishes were placed in a modular incubator chamber in aerobic conditions, gassed with 5% CO2 and 95% O2, and then incubated for 24 hours at 37 °C. After this period, a clean pipette tip was used to transfer each biopsy sample from the dish into a pre-labelled cryovial, minimizing sample contact and risk of contamination. The samples were immediately placed on ice (4 °C), flash-frozen in dry ice, and stored at −80 °C for long-term preservation before downstream analyses. The study workflow is shown in **Figure 1**.

### 2.5 Western blot analyses of human colon biopsies

Western blot analysis on human colon biopsies was performed loading 20 µg of total protein extracts for each sample on 10% polyacrylamide gels and transferred onto a PVDF membrane. The membranes were blocked in 5% non-fat dry milk for 1 hour and then were incubated overnight with the following primary antibodies: anti-COX2 (Cell signaling Technology, Cat. #5880) 1:1000, anti-CD54 (Elabscience, Cat. E-AB-31758) 1:1000 and anti-β-Actin (Elabscience, E-AB-20031) 1:5000. After a triple wash, membranes were incubated for 1 h with the HRP-Conjugated secondary antibodies: anti-rabbit IgG (Invitrogen, Cat. #31463) or anti-mouse IgG (Invitrogen, Cat. #31430) diluted at 1:10.000. Bands were visualized with ECL reagent (Thermo Fisher Scientific, Cat. #32209) according to the manufacturer’s instructions. Immunoblotting data were analyzed using ImageJ software to determine optical density (OD) of the bands. The OD readings of proteins were expressed as a ratio relative to β-Actin.

### 2.6 qPCR analysis of *in-situ* inflammatory factors

Real Time quantitative PCR was used to evaluate the gene expression of factors involved in inflammatory processes. mRNA was extracted from human colon biopsies collected at the end of treatment. The extraction was performed according to the standard TRIZOL Reagent protocol (Ambion by Life technologies cod.15596018). Once obtained, the mRNA is proceeded with the reverse transcription using the SuperScript VILO MasterMix (Invitrogen by Thermo Fisher Scientific cod.11755-050).

The cDNA obtained from the reaction has been amplified using the MicroAmp Fast Optical 96-well Reaction Plate with Barcode (0,1mL) (Applied Biosystems cod. 4346906) with iTaq Universal SYBR Green Supermix (BIORAD cod. #1725121) using primers reported in **Table 2**. The primer sequences used for qPCR analysis are shown in **Table 2**. qPCR was performed using an StepOnePlus™ Real-Time PCR System (Applied Biosystems).

**Table 2:** Primer sequences used for quantitative real-time PCR analysis.

Human target	Forward primer (5′→3′)	Reverse primer (5′→3′)
**IL-1β**	5′-GCACGATGCACCTGTACGAT-3′	5′-CACCAAGCTTTTTTGCTGTGAGT-3′
**IL-4**	5′-CCGTAACAGACATCTTTGCTGCC-3′	5′-GAGTGTCCTTCTCATGGTGGCT-3′
**IL-6**	5′-AGTTGCCTTCTTGGGACTGATG-3′	5′-TCCACGATTTCCCAGAGAAC-3′
**IL-13**	5′-CAATGGCAGCATGGTATGG-3′	5′-ATCCTCTGGGTCTTCTCG-3′
**IL-17**	5′-TCAACCGGATTGTCCACCAT-3′	5′-GAGTTTAGTCCGAAATGAGGCTG-3′
**IL-23**	5′-GAGCCTTCTCTGCTCCCTGATA-3′	5′-GACTGAGGCTTGGAATCTGCTG-3′
**IL-33**	5′-TGGAGGATGAAAGTTATGAG-3′	5′-TCAGGGTTACCATTAACATC-3′
**IFN-γ**	5′-GAGTGTGGAGACCATCAAGGAAG-3′	5′-TGCTTTGCGTTGGACATTCAAGTC-3′
**GAPDH**	5′-AAGGTGAAGGTCGGAGTCAA-3′	5′-AATGAAGGGGTCATTGATGG-3′

### 2.7 Immunofluorescence of colon biopsies and macrophages analysis

Immunofluorescence staining was performed on frozen sections (6μm) of human colon biopsies. After air-drying at room temperature, sections were fixed with 2% paraformaldehyde for 20 minutes and then washed with PBS 1X. Subsequently, they were permeabilized with 0.06% of Triton X-100 solutions and blocked with 3% BSA, 1% buffer solution (composed of: 0.02% di sodium azide, 50 mM di ammonium chloride, 0.005% di saponin and 0.5% BSA) 2.5% goat serum and 2.5% horse serum in PBS 1X for 30 minutes. Colon sections were incubated overnight at 4 °C with the following primary antibodies: mouse monoclonal anti-CD68 antibody (1:50, Abcam, catalog ab955, clone KP1), rabbit polyclonal anti-CD163 antibody (1:50 ABclonal, catalog A8383), and mouse monoclonal anti-CD206 antibody (1:50, Proteintech, catalog 60143-1). Colons were washed with PBS 1X and incubated for 1 hour with the following secondary antibodies: DyLight 549–conjugated anti- rabbit IgG (Vector Laboratories, catalog DI-1549) and Alexa Fluor 488–conjugated anti-mouse IgG (Vector Laboratories, catalog DI-2488). Nuclei were stained with Dapi. Images were acquired using a confocal laser-scanning fluorescence microscope TCS SP5 (Leica Microsystems).

### 2.8 Statistical analysis

Two-way ANOVA was used to analyze experiments in which two experimental factors were systematically varied group (Healthy vs. UC) and treatment (baseline, ω-3 lysine salt dispersion, SynΩ3, and SynΩ3 at different doses) allowing simultaneous assessment of the main effect of group, the main effect of treatment, and the group treatment interaction. For each cytokine and treatment condition, normality was assessed on the residuals of the two-way ANOVA model using the Shapiro-Wilk test combined with visual inspection of Q-Q plots, in accordance with recommendations (26). Two-way ANOVA was applied when residuals did not significantly deviate from a normal distribution. A p-value of less than 0.05 was considered statistically significant. Statistical analyses were performed using GraphPad Prism software (version 8.0, GraphPad Software).

## 3 Results

### 3.1 Characterization of SPMs produced by SynΩ3 formulation

**Figure 2:**
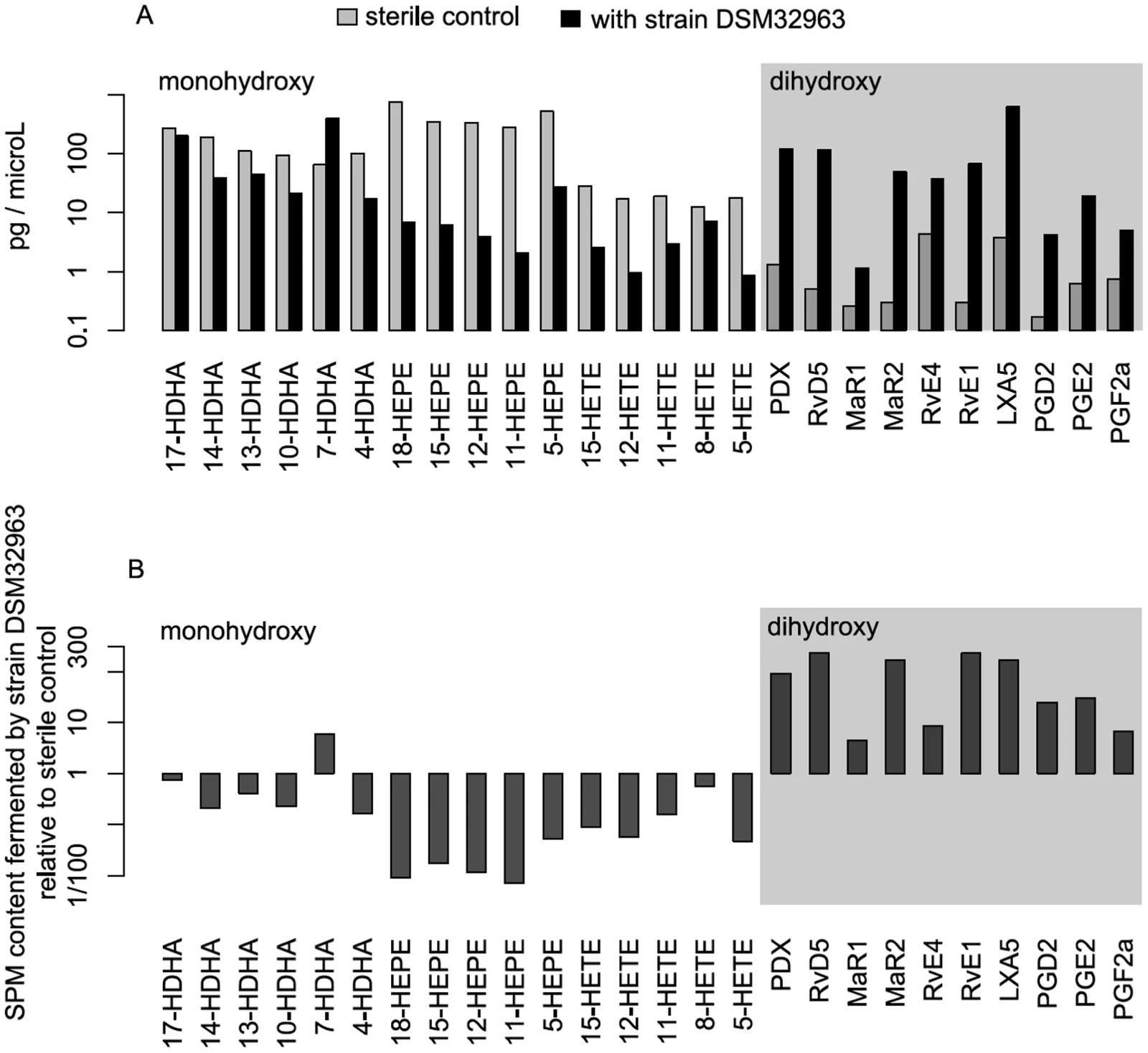
Conversion of EPA and DHA into specialized pro-resolving mediators (SPMs) by *B. megaterium* DSM 32963. **(A)** Absolute values of SPMs measured after incubation in the absence (sterile control) and presence of strain *B. megaterium* DSM 32963 (with strain DSM32963). **(B)** relative SPMs levels after fermentation with *B. megaterium* DSM 32963 relative to sterile control (calculated as [with strain DSM32963]/[sterile control]).

In the sterile control (ω-3 PUFA-lysine-salt only) we observed higher levels of monohydroxy DHA 17-HDHA, 14-HDHA, 13-HDHA, 10-HDHA,7-HDHA, 4-HDHA and EPA derivatives 18-HEPE, 15-HEPE, 12-HEPE, 11-HEPE, 5-HEPE, 15-HETE, presumably resulting from spontaneous, non-enzymatic oxygenation of the substrates. These monohydroxy derivatives accumulated but generated relatively low levels of di- and trihydroxy derivatives. In contrast, upon fermentation with B. megaterium DSM 32963, the pool of monohydroxy derivatives was further converted into the di- and trihydroxy ω-3 PUFA derivatives. This addition of B. megaterium DSM 32963 lead to up to 100 -fold increased levels of protectin PDX, resolvins RvE4, RvE1, RvD5 and lipoxin LXA5, and maresins MaR1, MaR2 (see **Figure 2**). This confirmed previous studies showing that B. megaterium DSM 32963 converts ω-3 PUFAs into a broad spectrum of resolvins, lipoxins, maresins & protectins via a microbial route independent from mammalian oxygenases and reductases. Further, the ferment also contained ~1.7% arachidonic acid and its derivatives (12-HETE, 11-HETE, 8-HETE, 5-HETE, PGD2, PGE2, PGF2a) and linoleic acid derivatives (9-HODE and 13-HODE).

### 3.2 IBD biopsies exhibit an exacerbated inflammatory profile compared to healthy tissues

**Figure 3:**
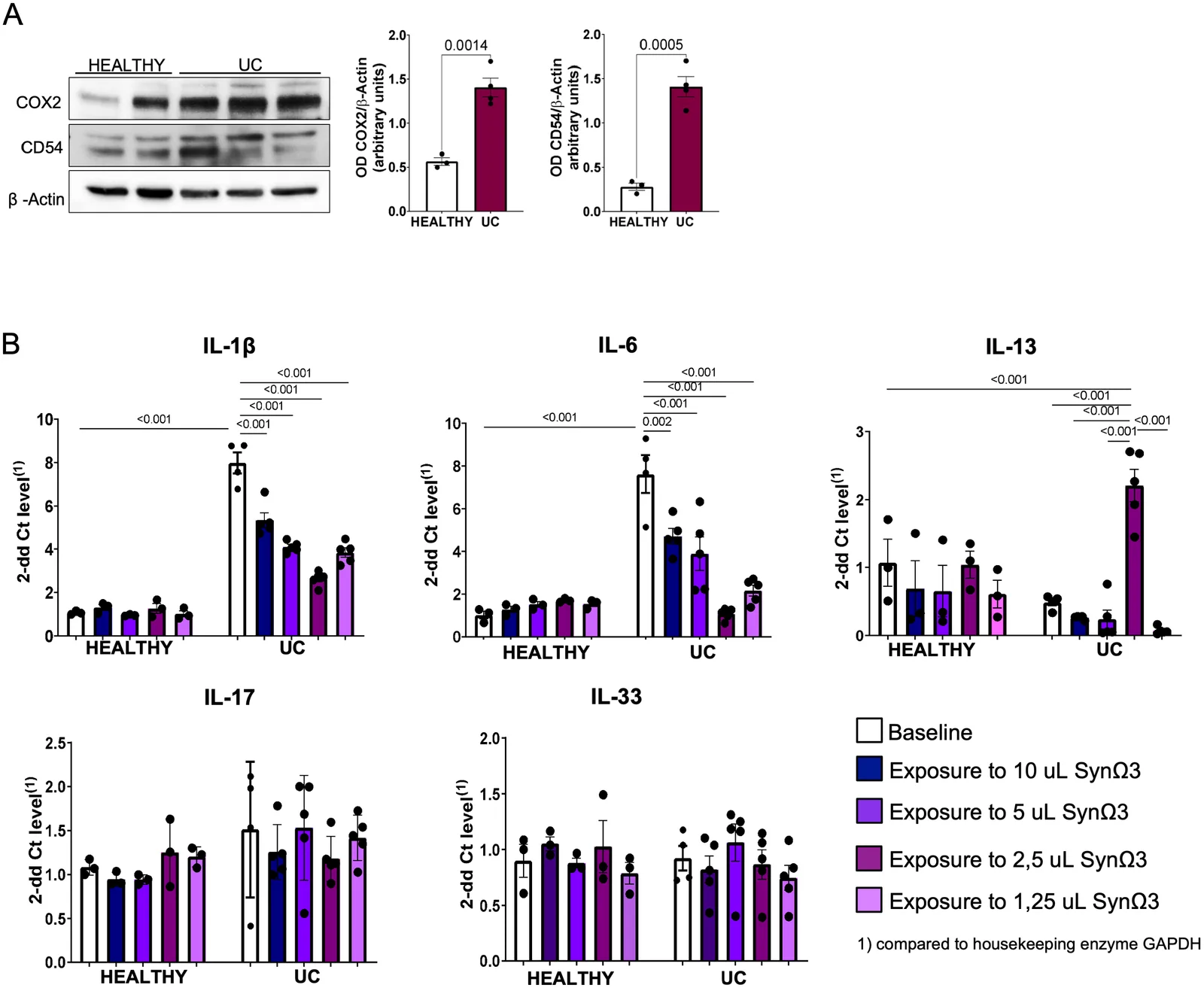
**(A)** Representative immunoblots and densitometric analyses of 3 independent experiments evaluating protein levels of COX2 and CD54, and β-Actin used as loading in human colon sections obtained from healthy and UC patients. *n* = 3 healthy donors and *n* = 4 UC donors. *p<0.05. **(B)** mRNA expression of IL-1b, IL6, IL13, IL17 and IL33 determined by quantitative reverse transcription polymerase chain reaction in human colon sections obtained from healthy and IBD patients treated with different dosages of SynΩ3 formulation (10, 5, 2,5 and 1,25mL) for 24 hours after collection. n = 15 healthy and n = 24 UC donors* p<0,005 vs all treatments within IBD group; # p<0,005 vs basal Healthy.

Initially, we compared the inflammatory profiles of intestinal biopsies from healthy subjects and patients with ulcerative colitis (UC). Our analysis revealed that the expression levels of cyclooxygenase-2 (COX-2) and intercellular adhesion molecule-1 (ICAM-1/CD54), two well-established markers associated with mucosal inflammation and leukocyte trafficking, were markedly upregulated in UC biopsies compared with healthy controls. These findings further support the presence of a sustained pro-inflammatory microenvironment, confirming the exacerbated inflammatory state characteristic of UC tissues (**Figure 3A**).

Once the pro-inflammatory condition had been established in patients with UC compared with control subjects, the efficacy of ex vivo treatment with SynΩ3 (Synbiotic B. megaterium DSM 32963/ω-3 PUFA)-derived ferments was subsequently investigated. Various concentrations of SynΩ3-derived ferments were tested on colon biopsies by analyzing the cytokine profile. As expected, our results revealed that colon biopsies obtained from UC patients in basal condition have higher levels of inflammatory interleukins IL-1β and IL-6 mRNAs than those obtained from healthy patients (**Figure 3B**). Interestingly, the treatment performed at different doses (10μL, 5μL, 2,5μL and 1,25μL) of SynΩ3-derived ferments for 24 hours modulated the expression of these mRNAs (**Figure 3B**). Specifically, at the dosage of 2,5μL, SynΩ3-derived ferments were able to modulate the relative expression of IL-1β and IL-6 in UC tissues, while biopsies obtained from healthy subjects revealed no modifications (**Figure 3B**). In addition, although the treatment of UC patients’ biopsies did not modify IL17 and IL33, we discovered a significant increase of IL13 by the dose of 2,5μL.

### 3.3 SynΩ3 -derived ferments modulate the cytokine profile in UC biopsies

To investigate the immunomodulatory effects of the treatments, we assessed the mRNA expression levels of key pro- and anti-inflammatory cytokines in colonic biopsies from both healthy individuals and UC patients. Quantitative qPCR analysis, normalized to the GAPDH housekeeping gene, revealed a distinct cytokine signature associated with UC and a significant response to the experimental treatments.

At baseline, UC biopsies exhibited a significantly higher expression of pro-inflammatory cytokines, including IL-1β, IL-6, IL-23, and INF-γ, compared to healthy controls. While treatment with ω-3 fatty acids alone resulted in a partial reduction of these markers, the exposure to SynΩ3-derived ferments exerted a potent synergistic effect (p-value <0.05).

**Figure 4:**
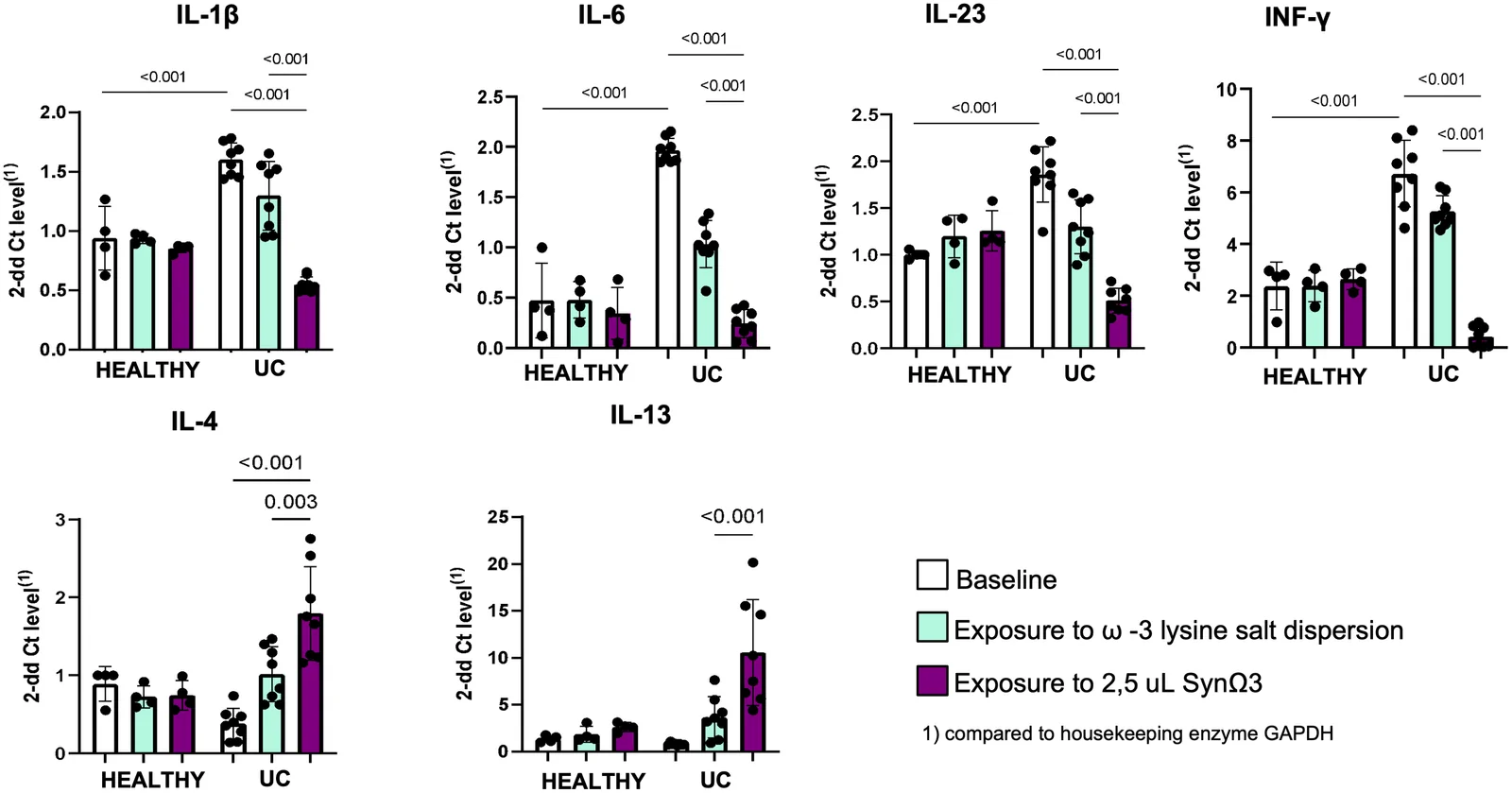
mRNA expression of IL-1β, IL-6, IL-4, IL-13, IL-23 and INF-γ determined by quantitative reverse transcription polymerase chain reaction in human colon sections obtained from healthy and IBD patients treated with the dosages of 2,5 μL of SynΩ3 formulation or ω-3 lysine salt dispersion alone for 24 hours after collection. *n* = 12 healthy and *n* = 24 UC donors * p<0,005 vs all treatments within IBD group; # p<0,005 vs basal Healthy.

Next, we evaluated the expression of the anti-inflammatory and pro-resolving cytokines IL-4 and IL-13. Interestingly, the treatment with SynΩ3-derived ferments significantly upregulated the mRNA levels of both cytokines in UC biopsies (p-value <0.05) (**Figure 4**).

### 3.4 The treatment with SPMs obtained from SynΩ3 formulation polarizes macrophages towards M2 pro-resolving phenotype in human colon biopsies

**Figure 5:**
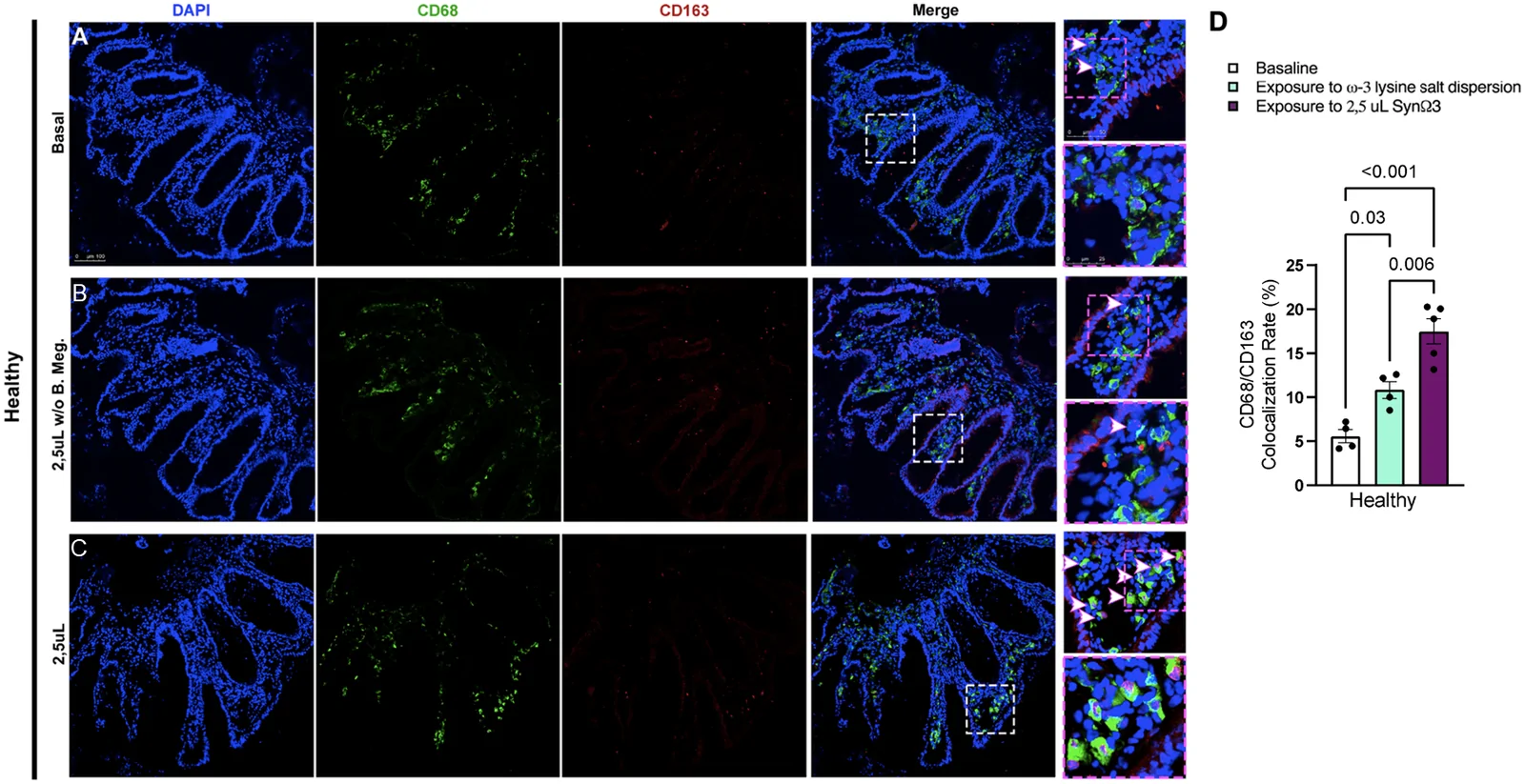
Representative images of immunofluorescence staining with CD68 and CD163 of human colon sections obtained from healthy patients in basal condition **(A)**, treated for 24 hours with 2,5 μL of SynΩ3 formulation **(B)**, or with ω-3 lysine salt dispersion alone **(C)**. DAPI-stained nuclei are in blue. Scale bar: 100μm; zoom inset up: 50μm; zoom inset down: 50μm. **(D)** The bar graph shows the CD68 and CD163 co-localization rate of human colon sections obtained from IBD patients in basal condition treated for 24 hours with 2,5 μL of SynΩ3 formulation or with ω-3 lysine salt dispersion alone. *n=*2.

**Figure 6:**
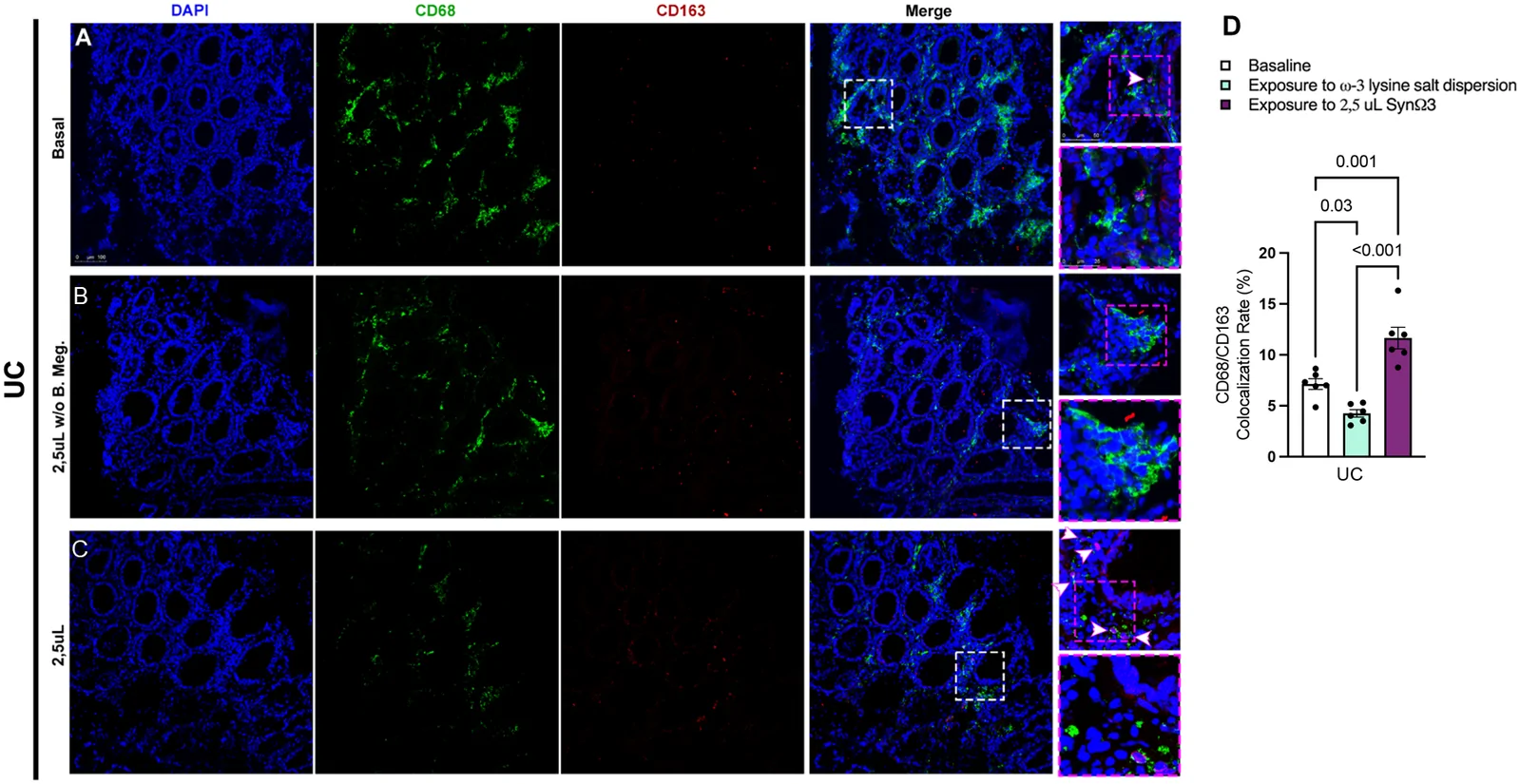
Representative images of immunofluorescence staining with CD68 and CD163 of human colon sections obtained from UC patients in basal condition **(A)**, treated for 24 hours with 2,5 μL of SynΩ3 formulation **(B)** or with ω-3 lysine salt dispersion alone **(C)**. DAPI-stained nuclei are in blue. Scale bar: 100μm; zoom inset up: 50μm; zoom inset down: 50μm. **(D)** The bar graph shows the CD68 and CD163 co-localization rate of human colon sections obtained from UC patients in basal condition treated for 24 hours with 2,5 μL of SPMs generated by SynΩ3 formulation or with ω-3 lysine salt dispersion alone. *n=* 3.

Previous studies have established that macrophages play a crucial role in the pathogenesis and resolution of UC, with M1-M2 phenotype polarization emerging as a central driver of this process (7). In line with this, our data demonstrate that ex vivo treatment with SPMs obtained from a SynΩ3 formulation successfully modulates the macrophage balance toward an anti-inflammatory phenotype. Moreover, our analysis of both healthy and UC colon biopsies confirms lower levels of inflammation in healthy tissue compared to UC tissue (**Figures 5**, **6**). Interestingly, in both conditions, ex vivo treatment with SPMs obtained from SynΩ3 formulation increased the number of double (CD68/CD163)-positive macrophages compared to biopsies treated with ω-3 PUFA lysine salt dispersion alone (**Figures 5B, C**, **Figures 6B, C**). This finding was further supported by the co-localization of CD163^+^/CD206^+^compared with both basal and the ω-3 PUFA lysine-salt control in biopsies from healthy participants and patients with UC, which indicates a modulation of macrophage-related markers towards an M2-polarized profile (**Supplementary Figures 1, 2**).

## 4 Discussion

This pilot ex vivo study provides preliminary evidence that the SynΩ3-derived ferment obtained by incubating *B. Megaterium* DSM 32963 with ω-3 PUFA lysine salt modulates the inflammatory response in human colonic biopsies. In biopsies from patients with ulcerative colitis (UC), the SynΩ3-derived ferment produced a greater reduction in the mRNA expression of the pro-inflammatory cytokines IL-1β, IL-6, IL-23, and IFN-γ than the ω-3 PUFA lysine-salt control. Treatment was also associated with increased expression of cytokines IL-4 and IL-13 and with an increased proportion of CD68^+^/CD163^+^ cells.

No statistically significant modulation of the evaluated cytokines was detected in healthy biopsies, whereas CD68^+^/CD163^+^ co-expression increased in both healthy and UC biopsies, suggesting that the cytokine response may be influenced by the basal inflammatory state of the tissue, while the macrophage-associated effect appears not to be restricted to diseased mucosa.

Collectively, these findings are consistent with the induction of a more resolution-associated tissue environment.

Inflammatory bowel disease (IBD) frequently develops in adolescence or early adulthood and generally requires long-term or lifelong clinical management. Due to the long duration of the condition, it can affect almost all parts of the gastrointestinal tract, as well as several other areas of the body, including the blood vessels, liver, kidneys, joints, eyes and skin (27).Although the etiology and pathogenesis of this condition remain enigmatic, several studies have demonstrated that the alteration of immunologic functions significantly contributes to the mucosal inflammation and disease progression (28). Over the last two decades, scientific evidence has demonstrated that circulating monocytes migrate to the inflamed colonic mucosa, where they differentiate into macrophage populations able to produce high levels of inflammatory interleukins, including IL-6, IL-1β, IL-23 and IFN-γ, and releasing reactive metabolites of oxygen and nitrogen and proteases that degrade the extracellular matrix and amplify the local inflammatory response (29).

Consistent with this inflammatory background, the present study showed that untreated biopsies derived from UC patients displayed higher expression of IL-1β, IL-6, IL-23, and IFN-γ than healthy biopsies. Increased expression of CD54 and COX-2 further supported the presence of a sustained inflammatory mucosal environment. Intercellular adhesion molecule-1 (CD54/ICAM-1) facilitates leukocyte-endothelial cell adhesion during inflammatory responses (30, 31), while cyclooxygenase 2 (COX2) contributes to the prostanoid biosynthesis during intestinal inflammatory responses (32). Together, these findings indicate that the biopsies used in the present study have molecular characteristics of UC-associated mucosal inflammation.

The present findings are consistent with previous experimental evidence identifying specialized pro-resolving mediators (SPMs) as active regulators of inflammation resolution. Rather than acting solely as conventional anti-inflammatory agents, SPMs regulate leukocyte trafficking, modulate inflammatory cytokine networks, and support the restoration of tissue homeostasis (33, 34).

In several experimental models of intestinal inflammation, exogenous administration of SPMs has exerted protective effects by reducing pro-inflammatory cytokine production (such as TNF-α, IL-1β, and IL-6), limiting leukocyte recruitment, and promoting inflammation-resolution pathways (35–37).

These observations provide biological plausibility for the reduction in IL-1β and IL-6 observed following exposure to the SynΩ3-derived ferment.

Nevertheless, using isolated SPMs as therapeutic agents directly presents several challenges, as they are unstable molecules that are rapidly metabolized by ubiquitous dehydrogenases and reductases, which limits their durability (38). For these reasons, several more stable synthetic analogues of SPMs and SPM receptor agonists have been generated to replicate the bioactivity of SPMs with improved durability and more straightforward synthesis (39–42).

Numerous studies have investigated conventional ω-3 PUFA supplementation for the modulation of chronic inflammation and cardiovascular risk. However, clinical trials have reported considerable variability in treatment responses. This heterogeneity has been attributed to differences in dose, supplementation duration, baseline ω-3 PUFA intake, formulation, bioavailability, age, sex, health status, and concomitant treatments (43–45). In this context, we adopted a synbiotic strategy designed to enhance the “endogenous-like” generation of SPMs through the enzymatic conversion of an ω-3 PUFA lysine salt by *Bacillus megaterium* DSM 32963. In a previous randomized study in healthy participants, SynΩ3 supplementation increased circulating levels of SPMs precursors compared with fish oil containing a similar amount of ω-3 PUFAs (46). These results support the ability of this formulation to influence the systemic SPM profile. Nevertheless, that study did not directly demonstrate intestinal SPM production or mucosal exposure.

Complementary evidence demonstrated that *B. megaterium* DSM 32963 can propagate and enzymatically convert ω-3 PUFAs into a broad spectrum of SPMs (resolvin E2 and protectin DX) and their precursors under simulated large-intestinal conditions compared with the fatty-acid formulation alone (47).

Although this study confirmed bacterial conversion under simulated colonic conditions, neither persistent propagation nor the production of SPMs has been demonstrated in UC patients *in vivo*.

In this context, our study aimed to evaluate the acute biological activity of a SynΩ3-derived ferment in human ex vivo mucosal tissue affected by ulcerative colitis, complementing the previous findings. The human supplementation study demonstrated systemic changes in several SPMs; the simulated large-intestinal model confirmed bacterial propagation and its use in producing SPMs from ω-3 PUFAs; and the present study provides preliminary evidence of acute biological activity in human colonic tissue ex vivo. Nevertheless, the present experiments do not identify which individual lipid mediator, or combination of mediators, is primarily responsible for the observed effects.

Moreover, a deeper analysis has revealed that the treatment was able to increase the levels of IL4 and IL13, two closely related cytokines that regulate immune pathways and can influence macrophage activation (48, 49). Previous studies have suggested that a shift from a pro-inflammatory M1-like state toward a more inflammation-regulating M2-like state may attenuate experimental colitis through modulation of cytokine networks and contribute to the resolution of mucosal inflammation (50, 51). However, the biological effects of IL-4 and IL-13 in the intestinal mucosa are context-dependent, and their increased expression should not automatically be interpreted as definitive evidence of an anti-inflammatory or pro-resolving response. Similarly, CD68/CD163 co-expression does not identify a homogeneous macrophage population or demonstrate functional reprogramming. After 24 hours of ex vivo exposure, the SynΩ3-derived ferment significantly increased the proportion of CD68^+^/CD163^+^ cells in colonic biopsies from both healthy participants and patients with UC, whereas the ω-3 PUFA lysine-salt control did not significantly alter the proportion of double-positive cells. Additional immunofluorescence analysis showed that SynΩ3 increased CD163/CD206 co-localization in both healthy and UC biopsies, further supporting treatment-associated modulation of macrophage-related markers. However, these findings remain compatible with an M2-like or pro-resolving-associated profile and do not demonstrate definitive functional macrophage reprogramming.

### 4.1 Study limitations

This study, conducted on ex vivo human colon biopsies, has enabled us to investigate the direct acute effect of SynΩ3-derived ferment on the mucosal microenvironment, whilst preserving the native multicellular architecture, which cannot be fully replicated in isolated cell models. Although this is the study’s key strength, several limitations should be considered when interpreting the present findings.

First, this was an exploratory pilot study involving a relatively small cohort of 13 patients with UC and six healthy controls. The sample size thus limits the statistical power but was sufficient to draw preliminary conclusions.

Second, the experiments were conducted using an acute 24-hour ex vivo biopsy model. Although this approach preserves the cellular architecture and local immune microenvironment of human intestinal mucosa, it cannot reproduce systemic immune regulation, intestinal transit, microbial competition, epithelial exposure over time, or host metabolism. Therefore, the present study does not provide information regarding gastrointestinal survival of the formulation, intestinal colonization by *B. megaterium*, mucosal bioavailability, pharmacokinetics, long-term safety, or clinical efficacy.

Third, macrophage characterization was based primarily on CD68 and CD163 or CD206 co-expression. These markers are compatible with an M2-like or pro-resolving-associated phenotype but do not establish macrophage functional reprogramming. Functional assays and broader phenotypic profiling should be performed.

Finally, SynΩ3-derived ferment contains a complex mixture of lipid mediators. Although metabololipidomic analysis has identified various resolvins, maresins, protectins, lipoxins and their precursors, we cannot be certain of the specific contribution of individual mediators or the possible interactions between them.

### 4.2 Future perspective

Future studies should validate these findings in larger, clinically characterized cohorts, including patients with UC at different stages of disease activity. Mechanistic studies using intestinal macrophages will help to understand whether the SynΩ3-derived ferment directly modulates macrophage function and pro-resolving signaling pathways. Subsequent *in vivo* investigations should assess safety and efficacy of SynΩ3 in UC and related diseases.

### 4.3 Conclusion

Overall, these findings support the potential of SynΩ3 to foster a less inflammatory, resolution-associated mucosal environment. SynΩ3 may therefore constitute a promising nutritional adjunct for UC, while its relevance to other IBD subtypes and chronic inflammatory conditions, including cardiovascular disease, warrants further investigation.

## Data Availability

The raw data supporting the conclusions of this article will be made available by the authors, without undue reservation.
